# A meta-theoretical framework for organizing and integrating theory and research on motivation for health-related behavior

**DOI:** 10.3389/fpsyg.2023.1130813

**Published:** 2023-02-23

**Authors:** David M. Williams

**Affiliations:** Department of Behavioral and Social Sciences, Center for Health Promotion and Health Equity, Brown University School of Public Health, Providence, RI, United States

**Keywords:** motivation, dual-processing, craving, desire, intention, goals

## Abstract

The concept of motivation is broad and multi-faceted. In health psychology, motivation has been conceptualized as cravings, urges, or desires for unhealthy behaviors, such as consumption of alcohol, cigarettes, or calorie-dense foods; or as behavioral intentions or proximal goals for healthy behaviors, such as smoking cessation, physical activity, or condom use. Because of the differences in terminology and associated theoretical underpinnings, it is difficult to characterize the state of the science or integrate research findings on motivation for health-related behavior. The present paper introduces a meta-theoretical Automatic-Reflective Motivation Framework (ARM-F) with the goals of organizing and integrating theory and research on motivation for health-related behaviors. At the first and broadest level, the ARM-F defines general motivation as *a wanting or desire to do something*. At the second level, it distinguishes between automatic and reflective motivation types, consistent with emerging perspectives in health psychology, historical and contemporary philosophical views on desire, and dual-processing perspectives in psychology. At the third level, the ARM-F preserves the nuanced terminologies and conceptualizations within the automatic (e.g., craving, urge, desire) and reflective (e.g., behavioral intention) motivation categories. The ARM-F has potential utility for organizing and integrating theory and research on motivation for health-related behavior, with implications for future research.

## 1. Introduction

One of the defining goals of health psychology is understanding the causal determinants of health-related behaviors, such as physical activity, eating, substance use, and sexual behavior. Among intrapersonal determinants of health-related behaviors, the extent to which someone is motivated to perform the behavior seems, intuitively, to be of paramount importance. That is, if we want to predict, understand, and change health-related behavior, we need to know whether people are motivated to be physically active, eat healthier, or abstain from addictive substances or high-risk sexual behaviors.

Among health psychology researchers, motivation is a murky concept. There are various constructs that potentially fall under the motivation umbrella, such as craving, urge, desire, behavioral intentions, and goals, but no agreed-upon higher-order definition of motivation. In fact, with the exception of researchers employing self-determination theory (SDT; Deci and Ryan, 1985), health psychology researchers tend not to even use the term *motivation*, instead employing theory-specific motivation labels and associated constructs. For example, in theories of addiction, motivation has been conceptualized as an affectively charged state of craving or urge that results from automatic associative processes (Robinson and Berridge, 1993; Koob and Le Moal, 1997; Kavanagh et al., 2005; Hofmann and Van Dillen, 2012). In contrast, in the context of socio-cognitive theories, motivation is conceptualized as a self-regulatory process that manifests in behavioral intentions or goals and leads to health-promoting behaviors such as healthy eating, regular physical activity, and avoiding addictive substances and risky sexual behavior (Fishbein, 1979; Rogers, 1983; Bandura, 1986; Ajzen, 1991).

The differences in theoretical perspectives and labeling of motivation concepts make it difficult to organize, accumulate, and integrate research on motivation for health-related behavior, despite the fact that so much work has been done. Indeed, in contrast to other health psychology constructs, such as self-efficacy and outcome expectancies, for which researchers working within specific health-behavior content areas have characterized and integrated findings across theoretical traditions (Ferrand et al., 2021; Newby et al., 2021; Bohlen et al., 2022; Pinquart and Borgolte, 2022), it is more difficult to characterize research on “motivation” in the broader field of health psychology or within specific content areas.^1^ Thus, despite the fact that motivation is clearly important to predicting, understanding, and changing health-related behaviors, the heterogeneity in motivation labels and theoretical approaches is a major barrier to synthesizing research on motivation within health-related behavior domains.

One promising development that could provide guidance for organization and integration of research on motivation in health psychology is the recent and intuitive proposal across multiple research groups that motivation can be divided into automatic and reflective types (Michie et al., 2011; Williams and Evans, 2014; Hofmann and Nordgren, 2015). For example, someone may intend to exercise because of the anticipated health benefits (i.e., reflective motivation), but also spontaneously experience dread in anticipation of their upcoming workout (i.e., automatic motivation). Likewise, someone may, without thought, experience craving for the chocolate cake at the dessert bar, but also want to avoid the cake and instead eat the more sensible fruit salad. This automatic-reflective distinction has the potential to provide a useful heuristic to acknowledge differences among diverse conceptualizations of motivation from the addiction (e.g., craving, urge) versus socio-cognitive (e.g., intentions, goals) traditions, while still placing them all under the same motivation umbrella, and thereby providing a platform for organizing and integrating research on motivation in health psychology.

**Figure 1:**
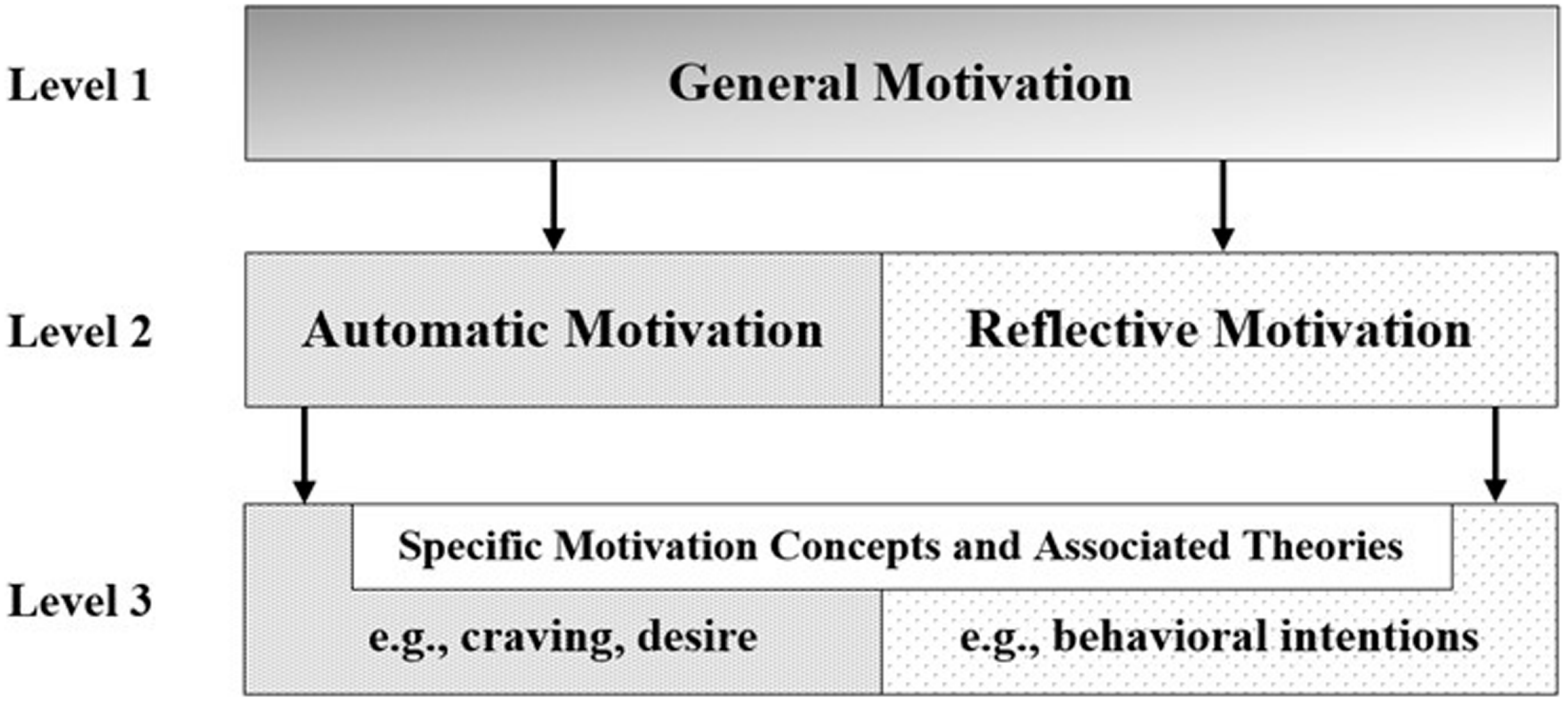
The automatic-reflective motivation framework.

The present paper parlays the automatic-reflective motivation distinction into a meta-theoretical automatic-reflective motivation framework (ARM-F) with the goals of organizing and integrating research on motivation across various theoretical traditions within health psychology. Specifically, the ARM-F consists of three hierarchical organizational levels: (a) a higher-order definition of motivation, (b) identification and labeling of automatic (craving, urge, and desire) and reflective (intentions and goals) motivation categories that fit within and thus can be integrated within the broader umbrella of motivation, and (c) preservation of nuanced but important distinctions among specific motivation concepts and associated theoretical traditions within the automatic and reflective motivation categories (Figure 1). The paper concludes with a discussion of the implications of the ARM-F for organizing and integrating research on motivation for health-related behavior, as well as the limitations of the framework.

The emphasis herein is on the distinction between automatic and reflective types of motivation, rather than an exhaustive review of all theories of motivation. Where specific theories of motivation are mentioned, they either (a) highlight the distinction between automatic and reflective motivation types, or (b) they provide an example of a theory of either automatic or reflective motivation that has been applied extensively in the context of health-behavior research.

## 2. Defining the general concept of motivation

In psychology, motivation has been defined broadly, with theories of motivation covering such concepts as energizing behavior, effort, persistence, and maintenance (e.g., Brehm et al., 1983; Wright, 2008; Heckhausen and Heckhausen, 2018). While these concepts are related to motivation, and are important for understanding what motivation does, motivation itself is more circumscribed.

In the Oxford online dictionary, motivation is defined, first, as “A reason or reasons for acting or behaving in a particular way” and, second, as “Desire or willingness to do something; enthusiasm” (Oxford University Press, 2022a). Similarly, (Baumeister, 2016), while asserting that, in general, “motivation is wanting” (p. 1), argues that there are “two meanings of motivation” (p. 2):

Many uses of the term motivation refer to broad, dispositional tendencies. Animals want food, safety, and sex. Crucially, they do not want these all the time, but in general, they show plenty of behavior designed to obtain these. Humans … likewise have desires for understanding and meaning, desires to be liked and respected, ambition, and other complex and advanced desires. These too are not constant urges but recurrent patterns. Motivation in this sense refers to recurrent patterns of desire and frequent behavioral tendencies. The person presumably does not feel this desire constantly nor continuously, nor does the person exhibit those behaviors on all occasions. And some people feel these desires more often and more intensely than other people.

The second concept of motivation refers to a particular desire to perform a particular behavior on a particular occasion. This second kind of motivation is thus highly instantiated and contextualized (i.e., it is here and now), and it is presumably characterized by subjective feeling of wanting something specific to happen—indeed, usually wanting to do something specific to make it happen.

Consistent with the second Oxford definition of motivation (Oxford University Press, 2022a) and Baumeister’s (2016) second of the “two meanings of motivation,” as well as his claim that “motivation is wanting,” motivation is defined herein as *a wanting or desire to do something*.

In contrast to motivation as defined above, the term *motive* is used herein to refer to the first Oxford (Oxford University Press, 2022a) definition of motivation (“A reason or reasons for acting or behaving in a particular way”), as well as Baumeister’s (Baumeister, 2016) first of the “two meanings of motivation” (see above). This use of the term motive, as distinct from motivation, is consistent with the Oxford definition of *motive* as “a reason for doing something” (Oxford University Press, 2022a), which is nearly identical to the first Oxford definition of *motivation*. The distinction between motivation and motive is illustrated in Table 1, Level 1. Motives (as opposed to motivations) may be physiological (e.g., hunger, thirst, libido; Hull, 1943) or psychological (e.g., achievement, affiliation, autonomy; McClelland, 1987; Ryan and Deci, 2017), with the former tending to vary more within persons and the latter between persons. Such motives may serve as partial determinants of motivation (e.g., Berridge and Aldridge, 2008; Battistelli et al., 2012), but are not a focus herein.

**Table 1:** Components of the automatic-reflective motivation framework.

*Level 1*: The general concept of motivation	General motivation is defined as *a wanting or desire to do something.* This definition of motivation is consistent with: - “Desire or willingness to do something; enthusiasm” (Oxford University Press, 2022a) - “motivation is wanting” (Baumeister, 2016) - “… a particular desire to perform a particular behavior on a particular occasion…” (Baumeister, 2016)	
*Level 2*: General motivation is divided into automatic and reflective motivation categories	Automatic motivations	Reflective motivations
Definitions	- Automatic affectively charged desires to perform or dread of performing a behavior that is associated with previous immediate pleasure (or reduced displeasure) or immediate displeasure (or reduced pleasure), respectively	- Affectively cold motivations to perform or not perform a behavior based on conscious and deliberate consideration of the outcomes of the behavior
Processing sources	- Automatic - Type 1 Processing - Based on associations with previous hedonic responses	- Controlled - Type 2 Processing - Based on deliberate consideration of future consequences of behavior
Psychological experience	- Experienced as affectively charged; an “appetite, hungering, craving, yearning, longing, [or] urge” - Occurring without any conscious and deliberate thought processes about the sources of one’s motivation - Difficult to articulate *reasons* for automatic motivations - Not possible to have second-order automatic motivations	- Experienced as affectively cold and without “appetite, hungering, craving, yearning, longing, [or] urge” - Involves conscious deliberation about the pros and cons of a given action or the outcomes of that action - Reasons for reflective motivations are constitutive of those motivational states - Possible to have second-order reflective motivations
*Level 3*: Examples of**s**pecific automatic and reflective motivation concepts	Automatic motivations	Reflective motivations
	- Craving - Urge - Desire	- Behavioral intention - Proximal goals

The present higher-order definition of motivation is necessarily broad and encompasses the specific motivation concepts including (but not limited to) craving, desire, urge, intentions and goals. This explicit and broad definition of motivation allows research on motivation from various theoretical traditions to be incorporated under one umbrella to facilitate characterization and integration of research on motivation in health psychology.

## 3. The distinction between automatic and reflective motivation

It has recently been proposed by multiple research groups that the general concept of motivation can be divided into automatic and reflective categories or types (Michie et al., 2011; Williams and Evans, 2014; Hofmann and Nordgren, 2015), as illustrated in Table 1, Level 2. In the context of the Capability-Opportunity-Motivation-Behavior (COM-B) model (Michie et al., 2011; Michie and West, 2013), Michie and colleagues state “With regard to motivation, we distinguished between reflective processes (involving evaluations and plans) and automatic processes (involving emotions and impulses that arise from associative learning and/or innate dispositions)” (Michie et al., 2011, p. 4). Likewise, in a subsequent publication, Michie and West argue, “Motivation is not simply a matter of reflective choice; a COM-B analysis requires an understanding of how reflective and automatic motivation interact to determine behavior (Michie and West, 2013, p. 7).

Similarly, in the affect and health-behavior framework (AHBF; Williams and Evans, 2014) motivation is divided into automatic and reflective types. “As defined by the AHBF, affectively charged [i.e., automatic] motivation for PA includes motivational states that have their basis in past affective responses to PA, such as craving, desire, dread, intrinsic motivation, and fear. Affectively charged motivation constructs differ from reflective motivation constructs, such as intentions and goals, which are a function of more deliberate consideration of the potential outcomes of a behavior” (Stevens et al., 2020).

A similar distinction between automatic and reflective motivation has also been made by Hofmann and colleagues (Hofmann et al., 2008, Hofmann and Van Dillen, 2012) application of the reflective-impulsive dual-processing framework (Strack and Deutsch, 2004) to the context of health behavior, in which “Desire can be defined as an affectively charged motivation toward a certain object, person, or activity that is associated with pleasure or relief from displeasure” (Hofmann and Van Dillen, 2012, p. 317; see also, Kavanagh et al., 2005). Though the authors’ original framework (Hofmann et al., 2008) did not emphasize the distinction between desire and more cognitively oriented forms of motivation, Hofmann and Nordgren (2015) later contrasted the concept of desire with cognitively derived goals—a position that is consistent with the distinction between automatic and reflective motivation, respectively.

The automatic-reflective motivation distinction is also similar to the dual-processing inspired want-should distinction in behavioral economics (Milkman et al., 2008; Bitterly et al., 2012), which generally maps on to automatic and reflective motivation, respectively. Finally, multiple dual motivational perspectives that are largely consistent with the automatic-reflective distinction have recently been posited in the context of physical activity behavior (Conroy and Berry, 2017; Brand and Ekkekakis, 2018; Stults-Kolehmainen et al., 2020).

Consistent with these previously made distinctions, automatic motivation is defined as an affectively charged desire to perform or dread of performing a behavior that is associated with previous immediate pleasure (or reduced displeasure) or immediate displeasure (or reduced pleasure), respectively. Reflective motivation is defined as an affectively cold motivation to perform or not perform or not perform a behavior based on conscious and deliberate consideration of the outcomes of the behavior (Table 1, Level 2).

### 3.1. Philosophical underpinnings of the automatic-reflective motivation distinction

The distinction between automatic and reflective motivation (Michie et al., 2011; Williams and Evans, 2014; Hofmann and Nordgren, 2015) can be traced back to the philosopher David Hume in his Treatise of Human Nature (Hume, 2000/1739). While Hume is famous for his distinction between reason and passion, he also argued that desires may be a function of either calm or violent passions, the former more deliberate and forward looking and the latter more a function of instinct (Radcliffe, 2015). Thus, the recent distinction made between reflective and automatic motivations (Michie et al., 2011; Williams and Evans, 2014; Hofmann and Nordgren, 2015) maps on to Hume’s distinction between calm and violent passions, respectively.

In contemporary philosophy, Davis (1982) similarly distinguishes between appetitive and volative desires:

In one sense, “desire” is synonymous with want, wish, and would like … I refer to desire in this sense as volative desire … In its second sense, “desire” has the near synonyms appetite, hungering, craving, yearning, longing, and urge … I refer to desire in this sense as appetitive desire (p. 181–182, emphasis in original).

Likewise, Vadas (1984) distinguishes between affective and non-affective desires:

"Desire" (as a noun) may refer to an affect, that is, a feeling, emotion, or mood, such as a desire to eat pizza, have children, or run in a marathon, and "to desire" (as a verb) is, in this sense, to be in a certain affective state, that is, one involving the feelings or affections; e.g., I desire to eat pizza, have children, or run in a marathon. This is to say that I feel, I am emotionally disposed toward, my eating pizza, my having children, my running in a marathon … But there is another sense of "desire, " a non-affective sense. In this sense I can be said to desire whatever goals I intentionally pursue (p. 277).

In recent automatic-reflective distinctions (Michie et al., 2011; Williams and Evans, 2014; Hofmann and Nordgren, 2015), automatic motivations are akin to Davis’s (1982) appetitive desires and Vadas’s (1984) affective desires, whereas reflective motivations are like Davis’s (1982) volative desires and Vadas’s (1984) non-affective desires.

### 3.2. Distinct cognitive sources of automatic and reflective motivations

The distinction between automatic and reflective forms of motivation implies differing cognitive sources that underlie automatic and reflective motivation as well as different experiential manifestations. According to dual-processing theory, there are two general types of cognitive information processing: automatic and controlled (Sloman, 1996; Smith and DeCoster, 2000; Strack and Deutsch, 2004; Gawronski and Bodenhausen, 2006; Evans, 2008; Kahneman, 2011). Automatic processing is more evolutionarily primitive, intuitive, based on associative learning, and occurs quickly and outside full conscious awareness except for the final output, which is often, though not always, consciously experienced. Controlled processing, on the other hand, is more evolutionarily recent, deliberative, based on rule-based learning, and occurs more slowly, with the subject conscious of the processing that leads to the final output (Evans, 2008).

Dual-processing theory has faced criticisms, particularly regarding division of all types of cognitive processing into just two (e.g., Osman, 2004; Kruglanski and Gigerenzer, 2011), and lack of empirical evidence that the two types of processing are perfectly aligned (i.e., always co-occur exclusive of the other type of processing), rather than merely correlated (e.g., Melnikoff and Bargh, 2018). However, proponents of the theory argue that its value is in recognizing the distinction between automatic and reflective types of processing regardless of whether or not each of these global types of processing includes further subdivisions or is merely correlated rather than perfectly aligned (e.g., Evans and Stanovich, 2013). Following from dual-processing theory, automatic motivations, such as cravings, urges, and desires, are based on automatic processing and are the mechanism through which previous immediate affective responses to behavior influence future behavior (Williams and Evans, 2014; Stevens et al., 2020). People automatically desire certain things, like alcohol and chocolate cake, and automatically dread other things, like grueling workouts and dental appointments, based on automatic associations with previous immediate affective responses to those behaviors. Automatic motivations are not a result of conscious expectations about the future pleasure of eating dessert or drinking another glass of wine, or the expected future pain and discomfort of an upcoming workout or root canal. This is not to deny that people do sometimes consciously expect pleasure or displeasure in response to a behavior (Kahneman and Snell, 1990; Mellers and McGraw, 2001; Conner, 2018). But such deliberate expectations of pleasure or displeasure are not necessary or sufficient to produce automatic motivations. One may think about tempting characteristics of the chocolate cake, such as its taste and texture, which may enhance craving for it (Kavanagh et al., 2005). Likewise, one may retrospectively infer a deliberate expectation of how good the cake would taste, which in turn led to automatic desire for the cake (Wood and Runger, 2016). However, automatic desire for the cake occurs without the need to think about the pleasure that will result from eating it or any reason for wanting it. That is, one need not think to themselves “the chocolate cake will taste so good” and therefore desire the cake. They just want the cake without having to think about it. Likewise, consideration of the future pain and discomfort of exercise is not necessary to automatically dread an upcoming workout. Instead, automatic motivations occur as a function of the immediate pleasure or displeasure that has resulted from past performance of the relevant behavior. That is, consistent with automatic processing in dual-processing theory, we have no choice in what we are automatically motivated to do in the same way we have no choice when we read 5 + 3 but to think about the number 8.

Conversely, reflective motivations, such as behavioral intentions and proximal goals, are posited to mediate the effects of beliefs about behavioral outcomes, social norms, and perceived capability on behavior (e.g., Bandura, 2004; Fishbein, 2008). Reflective motivations are a function of controlled and deliberate expectations regarding the consequences of our actions, including both affective and non-affective outcomes, and both immediate and more temporally distal outcomes. Thus, one source of reflective motivations is the expectation of pleasure or displeasure that we may experience in response to a behavior [i.e., anticipated affect, (Conner, 2018)]. But reflective motivations are also a function of expectations about the immediate consequences of behavior that are non-affective, such as our expectation that resisting the chocolate cake would be healthier, or our expectation that skipping our evening run would give us more time to spend with our kids before they go to bed. Reflective motivations may also be a function of more temporally distal outcomes or events that may be affective or non-affective. For example, we may be reflectively motivated to resist the chocolate cake because it will help us stick to our diet and ultimately lose weight. Likewise, we may decide to stick to our planned evening run because we want to maintain our new year’s resolution of running 3 days per week. Note that all potential outcomes of behavior (affective or otherwise) may be evaluated as good or bad, culminating in attitudes toward the target behavior (Ajzen, 1991) or outcome values (Bandura, 1997), and are thus still a function of evaluative affective processes. However, in contrast to the automatic processing of previous immediate affective responses to behavior (e.g., the pleasure of eating cake), what all of the factors that determine reflective motivations have in common is that they involve reflection or deliberation regarding the potential outcomes of a course of action (or inaction, e.g., skipping our planned exercise session). Thus, while it is the association with previous affective responses that underlie automatic motivations, reflective motivations may be a function of a broad variety of controlled processing inputs.

Importantly, automatic mental states may be consciously experienced, but are produced outside of consciousness, such as the experience of pain from stubbing one’s toe. An example of automatic desire is the desire for a fresh-baked cookie that one experiences upon smelling the cookies baking. If one has no intention or goal (i.e., reflective motivation) to lose weight or avoid sweets, then one may also form a conscious intention to go get a cookie. In this case, the automatic desire would be aligned with reflective desire. But they can, and often are, dissociated, such that one experiences an automatic desire to eat the cookie, but a reflective desire to avoid it. Automatic desires may be overcome in that one does not act on them; however, their automaticity means that one cannot directly control their production—that is, sometimes we have an uncontrollable hankering for things we would rather not want.

### 3.3. Distinct psychological experiences of automatic and reflective motivation

There are also differences in the psychological experience of automatic and reflective motivations. First, automatic motivations are, consistent with theories of craving and desire, affectively charged (Drummond, 2001; Kavanagh et al., 2005; Skinner and Aubin, 2010; Tiffany, 2010; Hofmann and Van Dillen, 2012; Sayette, 2016; Williams, 2018). Like Davis’s (1982) appetitive desires, automatic motivations are experienced as an “appetite, hungering, craving, yearning, longing, [or] urge.” Conversely, reflective motivations, like Davis’s (1982) volative desires, lack these experiential qualities and are instead more cold and calculated. Likewise, as indicated in their labels, Vadas (1984) distinguishes between affective and non-affective desires based on the affectively charged nature of the former but not the latter.

Second, automatic motivations feel as though they spring, automatically, from within. Thus, it is difficult to articulate *reasons* for automatic motivations. Indeed, it would seem peculiar and perhaps off-putting to ask someone who you know enjoys chocolate cake or beer “Why do you want the chocolate cake?” or “Why are you craving a beer?” One simply wants cake or beer—there are certainly causes for such automatic desires, but typically no reasons that one can easily articulate. Conversely, the reasons for reflective motivations, such as behavioral intentions are constitutive of those motivational states (1982; 1984). Thus, it makes sense and would be appropriate to ask someone who has just turned down the chocolate cake: “Why *don’t* you want the chocolate cake?” or “Why don’t you want a beer?” The desire to *not* have chocolate cake or beer is (or at least is more likely to be) a reflective desire that has reasons, such as wanting to cut back on sweets or alcohol, trying to lose weight, or needing to wake up early the next morning.^2^ Moreover, it is possible to have higher-order reflective motivations about our automatic motivations (Frankfurt, 1971). For example, one may reflectively desire not to want to drink (“I wish I did not *want* to drink so much”) or to want to exercise (“I want to *want* to exercise, but the fact is I just do not want to”).

### 3.4. Automatic and reflective motivational conflict

The distinction between automatic and reflective motivation is evident in the context of conflicts between automatic and reflective motivation types (West, 2007; Hofmann et al., 2008). For example, someone may want to quit smoking but at the same time desperately crave a cigarette. May want to exercise but also dread their upcoming workout. May crave the chocolate cake but also want to avoid the cake and instead eat the more sensible fruit salad. May want to obtain a colonoscopy but also dread the procedure. May desire immediate sex with a new partner but also want to wait until a time when contraception is available. May want to eat more vegetables, but also dread experiencing their taste and texture. May want one more drink at the bar but also want to stop drinking and retire for the night. Although there is variability across persons and contexts, in most cases the motivations to smoke a cigarette, skip the workout, eat the chocolate cake, cancel the colonoscopy, have sex without a condom, push away the vegetables, and have one more drink at the bar represent automatic motivations: what is wanted based on automatic behavior-affect associations, with no deliberate consideration of behavioral consequences. Conversely, the motivations to quit smoking, perform one’s planned workout, eat the fruit salad instead of the chocolate cake, proceed with the colonoscopy, forgo immediate sex, eat one’s vegetables, and refrain from another drink at the bar represent reflective motivations: what is wanted based on deliberate consideration—in the moment and/or at some previous time—of the consequences of one’s actions.

Such motivational conflicts have been previously discussed highlighted (West, 2007; Hofmann et al., 2008). The ARM-F highlights that it is automatic and reflective motivation types (Michie et al., 2011; Williams and Evans, 2014; Hofmann and Nordgren, 2015) that are often in conflict, while acknowledging that both types of motivation fit within the broader concept of motivation.

## 4. Specific automatic and reflective motivation concepts

While, for heuristic purposes, motivation may be categorized into automatic and reflective types, there are still important differences in motivation concepts within the automatic and reflective motivation categories (Table 1, Level 3). For example, motivation is explicitly posited to manifest as behavioral intentions in Protection Motivation Theory (PMT, Rogers, 1975, 1983), and the Theories of Reasoned Action and Planned Behavior (TRA and TPB, Fishbein, 1979; Ajzen, 1985); however, motivation is characterized as a self-regulatory process in the context of SCT, culminating in formation of proximal goals. Moreover, the cognitive antecedents of motivation in these socio-cognitive theories—while all a function of reflective processing—differ to some extent across theories.

Likewise, there are nuanced but important differences in conceptualizations of motivation within the category of automatic motivation. There is a rich tradition of theory and research on the craving concept, and not all approaches to craving are the same (Skinner and Aubin, 2010). For example, some theorists view urges as less extreme instances of craving, while others view these as discrete motivational states (Sayette, 2016). Such nuanced views on craving and urge should be preserved. Similarly, recent approaches to desire in the context of health psychology (Kavanagh et al., 2005; Hofmann and Van Dillen, 2012), while largely overlapping, nonetheless have important differences in emphasis that should be acknowledged.

## 5. Implications

The ARM-F is a meta-theoretical framework with the goals of organizing and integrating research on motivation. To date, the literature on craving, urge, and desire (i.e., automatic motivations) for health-related behaviors has been largely separate from the literature on behavioral intention and proximal goals (i.e., reflective motivations), consistent with the differences in the craving-desire versus socio-cognitive approaches to motivation for health-related behavior. Traditionally, researchers employing automatic motivation concepts are more interested in understanding causal determinants of tempting but unhealthy behaviors (e.g., addictive behaviors and unhealthy eating), and those employing reflective motivation concepts are more likely to be interested in understanding the determinants of successful self-regulation (e.g., abstaining from addictive behaviors, engaging in regular exercise). Moreover, as noted above, researchers studying craving, urge, desire, intention, or proximal behavioral goals tend not to frame or incorporate their findings within the broader context of motivation. Thus, theory and research on motivation for health-related behavior is divided across disparate literatures.

In addition to providing a single broad conceptualization of motivation, the ARM-F adopts the emerging consensus regarding the distinction between automatic (e.g., craving, urge, desire) and reflective (e.g., behavioral intentions and goals) motivation types (Michie et al., 2011; Williams and Evans, 2014; Hofmann and Nordgren, 2015) that is reflected in the disparate addiction and socio-cognitive approaches to motivation. While research in health psychology tends to emphasize either automatic or reflective motivation, consideration of both types of motivations is needed to improve understanding of unhealthy behavior and to facilitate change in the healthy behavioral alternative. Acknowledgment that automatic and reflective motivation are both types of the higher-level motivation constructs that can facilitate integration across theoretical approaches.

For example, the anti-reward theory of addiction (Koob and Le Moal, 2008a,b) posits that withdrawal symptoms trigger craving for addictive substances, which then lead to addictive behaviors, including tobacco smoking (Watkins et al., 2000). Reflective motivations, such as behavioral intentions, play no role in the anti-reward theory of addiction. Conversely, the TPB (Ajzen, 1991) posits that smoking cessation is a function of behavioral intentions and their belief-based antecedents (Lareyre et al., 2021), with no attention to the automatic motivational processes that maintain smoking behavior in the face of cessation attempts. In order to better predict, understand, and change smoking behavior, these perspectives may be usefully combined. The ARM-F facilitates integration of these two approaches such that one would expect both craving for cigarettes and intentions to quit smoking—examples of automatic and reflective motivations, respectively—to affect smoking behavior. Thus, the anti-reward theory of addiction and the TPB can be integrated in the context of smoking cessation research by incorporating both craving for smoking and intentions to quit within the same conceptual model.

Another example of the potential for theoretical integration in motivation research can be seen in the context of eating behavior. Incentive salience theory (Robinson and Berridge, 1993) has produced a voluminous literature on the palatability of foods (Berridge et al., 2010), emphasizing the distinction between ‘liking’ and ‘wanting’—the latter a form of automatic motivation. However, this literature on automatic motivation (i.e., ‘wanting’ or incentive salience) for foods is detached from the literature on eating behavior based on SCT (Bandura, 1986), which emphasizes the formation and pursuit of proximal goals for healthy eating (Guillaumie et al., 2010; Bagherniya et al., 2018). According to the ARM-F, both ‘wanting’ of foods, a form of automatic motivation, and proximal goals to avoid snacks or eat fruits and vegetables, a form of reflective motivation, may be usefully integrated in attempting to understand eating behavior. Thus, the incentive salience theory and SCT can be integrated in the context of eating research by incorporating both “wanting” of specific foods and proximal goals to eat those foods within the same conceptual model.

The ARM-F can be used as a basis for integrating theory and research on motivation for other health-related behaviors, including other addictive substances (besides nicotine; e.g., alcohol, opiate use) and behaviors (e.g., gambling), contraceptive use, exercise behavior, cancer screening, and medication adherence. Existing theory and research in each of these behavioral domains tends to emphasize either automatic motivation or reflective motivation. However, a complete understanding of the motivations for health-related behaviors must include attention to both types of motivation.

## 6. Limitations

The ARM-F is meta-theoretical framework, not a causal theory, and thus it does not propose specific hypotheses. Moreover, the ARM-F is inductive in nature, using existing ideas—including the previously proposed distinction between automatic and reflective motivation (Michie et al., 2011; Williams and Evans, 2014; Hofmann and Nordgren, 2015)—in an attempt to provide a scaffolding for organizing motivation concepts based on existing research from as yet disparate theoretical traditions. It does not propose to compete with other views of motivation, but rather to integrate diverse approaches under a single umbrella. Likewise, because the ARM-F is a meta-theoretical framework rather than a causal theory of behavior, it is agnostic as to how, exactly, automatic (e.g., desires) and reflective (e.g., intentions) interact to influence behavior (e.g., Perugini and Bagozzi, 2004; Hofmann et al., 2008; Papies, 2016).

The distinction between automatic and reflective motivation is not proposed to be a distinction between natural kinds. Motivation, as well as the delineation of automatic and reflective motivation types, are psychological constructs for which there are no natural kinds. However, the distinction between automatic and reflective motivation is highlighted herein because (a) it is consistent with a growing consensus (Michie et al., 2011; Williams and Evans, 2014; Hofmann and Nordgren, 2015), (b) it has roots in historical and contemporary academic philosophy (1982; Hume, 2000/1739; Radcliffe, 2015; 1984), and (c) it is consistent with dual-processing approaches in psychology and health psychology (Sloman, 1996; Smith and DeCoster, 2000; Strack and Deutsch, 2004; Gawronski and Bodenhausen, 2006; Evans, 2008; Hofmann et al., 2008; Kahneman, 2011).

Finally, the ARM-F does not attempt to supplant the previously articulated distinction between intrinsic and extrinsic motivation inherent to SDT (Deci and Ryan, 1985). Unlike other theoretical approaches often employed in health psychology research, motivation is clearly and precisely labeled and conceptualized in SDT, including multiple subtypes of motivation (Ryan and Deci, 2000; Ryan et al., 2009, 2021; Deci and Ryan, 2012). Moreover, because the term “motivation” is explicitly used, it is easier to identify and synthesize research on motivation conducted in the context of SDT relative to the socio-cognitive or craving-desire approaches. For example, a recent meta-analysis showed that autonomous motivation, but not controlled motivation or amotivation was predictive of health behaviors (Ntoumanis et al., 2021). However, while the various types of motivation in SDT may fit with the definition of general motivation in the first level of the ARM-F, the distinction between intrinsic (or more autonomous) and extrinsic (or more controlled) does not clearly map onto the distinction between automatic and reflective motivation or the conceptualizations of motivation within the addiction and social-cognitive traditions reflected in the latter distinction. Nonetheless, the ARM-F and SDT should not be viewed as competing theories of motivation, as the ARM-F is not a causal theory of motivation but a framework intended to organize and integrate theories of motivation. Indeed, attempts to reconcile the SDT approach to motivation within the ARM-F are welcome, as this would allow for integration of the SDT approach to motivation with the socio-cognitive and craving-desire approaches.

## 7. Summary and conclusions

The concept of motivation is broad and multi-dimensional. In health psychology, with the exception of those employing SDT, researchers rarely use the term *motivation* when studying the effects of craving, urge, desire, intentions, or goals on health-related behavior. Because of the different points of emphasis and different terminology in different approaches to motivation, it is difficult to characterize, integrate, and accumulate findings on the broader concept of motivation.

The present paper introduced the ARM-F with the purpose of organizing and integrating the literature on motivation for health-related behaviors. The ARM-F defines general motivation as *a wanting or desire to do something.* It distinguishes between automatic and reflective types of motivation, consistent with the addiction and socio-cognitive perspectives, with distinct cognitive sources and psychological experiences, while preserving the nuanced differences among theories of automatic motivation (e.g., various theories of craving and desire) and reflective motivation (e.g., SCT, PMT, TPB). The ARM-F provides a means for integrating existing and future research on diverse motivation concepts and thus facilitating a more complete understanding of motivation for health-related behaviors.

## Author contributions

The author confirms being the sole contributor of this work and has approved it for publication.

## Funding

This work was supported by grants to DW from the National Institute on Aging under grant number R01AG069349; the National Cancer Institute under grant number R01CA262894; and the National Center for Complementary and Integrative Health under grant number R34AT011302.

## Conflict of interest

The author declares that the research was conducted in the absence of any commercial or financial relationships that could be construed as a potential conflict of interest.

## Publisher’s note

All claims expressed in this article are solely those of the authors and do not necessarily represent those of their affiliated organizations, or those of the publisher, the editors and the reviewers. Any product that may be evaluated in this article, or claim that may be made by its manufacturer, is not guaranteed or endorsed by the publisher.

## Footnotes

^1^An exception is the specialized definition and operationalization of motivation within SDT, for which there have been summaries of research in health psychology (e.g., Gillison et al., 2019).

^2^Of course, some people just do not like chocolate cake and so the desire to not have the chocolate cake would be an automatic desire.
